# Anterior Distal Tibia Hemi-Epiphysiodesis in Relapsed Ponseti Treated Clubfoot Patients: Beware of the Rebound Phenomenon †

**DOI:** 10.3390/children13101362

**Published:** 2026-10-08

**Authors:** Arnold Besselaar, Isabelle van Tilburg, Marieke van der Steen

**Affiliations:** 1 Department of Orthopaedic Surgery & Trauma, Máxima Medical Center, 5600 PD Eindhoven, The Netherlands; 2 Department of Orthopedic Surgery, Care and Public Health Research Institute (CAPHRI), Maastricht University Medical Center+, 6229 AN Maastricht, The Netherlands

**Keywords:** clubfoot, relapse, equinus, anterior epiphysiodesis, 8-plate

## Abstract

**Highlights:**

**What are the main findings?**

• Anterior hemi-epiphysiodesis of the tibia in clubfoot patients with recurrent equinus show effective slope correction but in young children, also a rebound phenomenon.

**What are the implications of the main findings?**

• Patients and parents should be counselled about this side effect, and possibly the timing of the procedure has to be adapted to the age of the child.

**Abstract:**

**Background/Objectives**: Anterior hemi-epiphysiodesis of the distal tibia (AHDT) with an 8-plate has been proposed as treatment for clubfoot patients with recurrent equinus deformity. However, to date, clinical results have been inconsistent and particularly focused on correction during treatment. In the current project, we specifically added evaluation of outcomes at least one year after 8-plate removal. **Methods**: We included Ponseti treated idiopathic clubfoot patients aged between 4 and 12 years who underwent AHDT between 2015 and 2022. Patients were assessed preoperatively at indication for 8-plate removal and if available at least one year after 8-plate removal. A senior orthopaedic surgeon specialized in clubfoot treatment determined passive ankle dorsiflexion. Furthermore, on standardized lateral radiographs, the anterior distal tibial angle (ADTA) was measured. **Results**: The median treatment duration of 46 feet (32 patients, 38% female) was 22.1 months (95–CI: 19.3–24.9). Both ankle dorsiflexion and ADTA improved significantly during AHDT. Of 35 feet, at least 1 year of follow-up after removal of hardware was available. The ADTA showed a significant increase (*p* < 0.001) after 8-plate removal, by modelling to preoperative values. The measured ankle dorsiflexion one year after 8-plate removal showed no change compared with the pre-treatment situation. One patient undergoing bilateral AHDT experienced a wound infection and screw migration. **Conclusions**: AHDT using an 8-plate is an effective method to achieve slope correction. However, after 8-plate removal, rebound of ADTA can occur. Physicians, parents, and patients should be informed about this mechanism, which might result in different timing of the treatment and possibly the need for renewed AHDT.

## 1. Introduction

Treatment of idiopathic clubfoot can be complicated by a relapse tendency [1]. The Ponseti method is nowadays the preferred treatment for clubfoot, with good to excellent results [1,2,3]. However, percentages of relapses after Ponseti treatment in the literature vary significantly between 1.9 and 67% [4,5]. These relapse deformities include hindfoot varus, midfoot adduction, cavus and impaired dorsiflexion [6,7]. The most common relapse deformity is impaired ankle dorsiflexion, possibly responsible for the pain and functional limitation relapse patients encounter [6,7]. Patients experiencing a relapse show deviations in their gait pattern and score lower on both patient-reported and clinical outcome measures [8,9,10]. The decreased ankle dorsiflexion may be the result of a flat top talus, joint stiffness, soft tissue imbalance or posterior tightness [11,12].

Previously reported relapse treatment strategies to influence impaired dorsiflexion vary from repeated serial casting or Achilles tenotomy to extensive soft tissue release or corrective osteotomies [11,13]. These intra-articular surgical interventions are, however, associated with an increased risk of perioperative complications and prolonged recovery [13]. Another less-applied technique uses guided growth by means of a temporary anterior hemi-epiphysiodesis of the distal tibia (AHDT) with a small 8-plate. Due to this minimally invasive surgical procedure, the growth plate is partially inhibited through a tension band principle, resulting in a planned axis deviation [14]. As a consequence, a slight recurvatum of the distal tibia occurs, theoretically leading to a foot position in more dorsiflexion [14,15,16].

Limited literature is available on the results of AHDT with an 8-plate as relapse treatment for clubfoot patients with recurrent equinus deformity [15,16,17,18]. Often, these findings were based on small populations of initially surgically treated clubfoot patients. While some studies found both clinical and radiological benefits after hemi-epiphysiodesis in carefully selected groups of clubfoot patients [15,16,17], the effects of epiphysiodesis have not been unequivocally reported in the literature. In one study of 31 clubfeet treated with epiphysiodesis, no clinically significant improvement was mentioned [18]. No or only a few minor related complications were reported [15,16,17,18]. However, follow-up over a longer period is lacking. As a result, possible decline of recurvatum of the distal tibia after implant removal has scarcely been considered.

The aim of this study was to establish whether AHDT with 8-plate is a safe and effective treatment for sagittal residual deformity in clubfeet after Ponseti treatment. To this end, we radiologically and clinically evaluated patients preoperatively, at indication for 8-plate removal and, if available, at least one year after 8-plate removal.

## 2. Materials and Methods

This retrospective analysis of a single-centre cohort included data from children who visited the outpatient clinic of the tertiary clubfoot centre at Máxima MC with a relapsed clubfoot and who were treated with AHDT between 2015 and 2022. Approval of the Institutional Review Board was obtained [reference METC Máxima Medisch Centrum: 2014-67], and legal guardians gave their permission to collect and analyse this data.

### 2.1. Study Population

Patients with uni- or bilateral idiopathic clubfoot were included if they were initially treated with the Ponseti method and experienced a relapse of the sagittal residual deformity for which AHDT was indicated. A hemi-epiphysiodesis was indicated if dorsiflexion—passively tested—was less than 0 degrees. In addition, the so-called ‘end feeling’ had to be rigid as an indication that the Achilles tendon did not interfere with raising the foot. Furthermore, flattening of the talus seen on X-ray added to the indication for relapse treatment by means of AHDT. To be included in the study, removal of the 8-plate needed to be between January 2015 and December 2022, with an age not older than 12 years due to the limits of the cohort. The Anterior Distal Tibial Angle (ADTA) needed to be measurable on the pre-operative X-ray of the ankle, intra-operatively or within 3 months after implantation, before influence of guided growth was seen. Syndromic, neurogenic, or positional clubfoot were excluded. In children with bilateral clubfoot, each foot was considered separately.

### 2.2. Treatment

The procedure of implantation is performed in a supine position under general anaesthesia with a popliteal nerve block by a standard anterior approach. One 8-plate (Pro-Motion Medical^®^, Zwijndrecht, The Netherlands) is placed in the anterior centre of the distal tibia on the intact periosteum as advised [14]. Under image intensification, the centre of the tibia in the frontal plane is marked with a smooth Kirschner (K-) wire. Subsequently, the leg is rotated to perform a lateral image to place the K-wire in the epiphyseal part of the distal tibia. First, the distal screw and plate were mounted, then the proximal screw was placed. The wound is closed in layers. The post-operative regime includes immediate functional and full weight-bearing instructions; no plaster is used. Physiotherapy is prescribed if desirable.

Two weeks after AHDT, post-operative wound assessment took place. Guided growth was monitored by means of clinical follow-up with X-rays at 3 months and then every 6 months. The indication for removal was based on clinical and radiological grounds. If the children clinically showed a walking pattern with proper heel landing and/or a radiological posterior coverage with the tibial plafond at the talus or an ADTA change of 15–20 degrees was seen, material was removed [16,17]. As there is no research available on the late effects of AHDT of angles above 20°, we have chosen a safe margin, from 15 to 20°, as a criterion for removal. In higher angles, the tibiaplafond shifts relatively radiologically far posteriorly in relation to the talus.

Removal of the 8-plate was performed by a similar surgical procedure as implantation. All patients are included in our follow-up protocol till the end of growth. In addition to the standard check-ups according to age and treatment phase, children are assessed more frequently in the event of relapse treatments or new complaints.

### 2.3. Radiographic Measurements

The effect of AHDT was based on the difference in ADTA measured at the X-ray showing the starting point of treatment and the X-ray made at indication for removal of the 8-plate. Every clubfoot patient where flatting of the talus is expected because of a hard end-feeling of the dorsal flexion movement and/or restricted dorsiflexion (less than 10 degrees) is checked with a standardised X-ray. In addition, radiographically measured ADTA at least 12 months after implant removal was used to investigate whether the effect of epiphysiodesis was maintained. ADTA was measured on standard weight-bearing lateral X-rays of the ankle (Figure 1). The X-ray was defined as measurable if there was an overlap of the medial and lateral domes of the talar trochlea, and the distal fibula was superimposed by the tibia [19,20]. All radiographic measurements were performed by the same researcher (IT). To assess intra-rater reliability, a random sample of 19 X-rays was re-evaluated by the researcher. The blinded re-evaluation was executed at least three weeks after initial assessment of the X-ray.

### 2.4. Anterior Distal Tibial Angle

The ADTA is the angle between the shaft of the tibia and the tibial plafond [15,16,21]. The ADTA was determined at the intersection of the tibial shaft axis with a straight line through the posterior and anterior borders of the tibial plafond, as illustrated in Figure 2A. The midpoint of the tibial shaft and consequently the axis of the tibia were determined using two supporting lines. A normal value in non-clubfeet for ADTA is 80 ± 2 degrees [21,22].

### 2.5. Alpha Angle

The alpha angle is measured as an indication of flattening of the talus. This angle reflects the opening of the angle of the talar dome and is measured in the sagittal plane by means of a Mose ring spanning between the anterior and posterior trochlea (as illustrated in Figure 2B) [23]. A normal alpha angle in non-clubfeet is 117.9 ± 15.0 degrees [23]. A decrease in the alpha angle indicates more talar flattening, as illustrated in Figure 2C,D.

### 2.6. Dorsiflexion Measurement

Dorsiflexion with an accuracy of 5 degrees was assessed during outpatient clinic visits by an experienced paediatric orthopaedic surgeon specialising in clubfoot pathology (AB), using a goniometer. The measurement was taken from the medial side of the foot with the sole of the foot and the tibia shaft as reference. For every child in our clinic with clubfoot, we plan clinical check-ups at least every 6 months. The visits always include a physical examination (testing the range of motion of foot and ankle, determining leg length and individual muscle testing) and functional tests such as walking, running, hopping and stability testing. Scores are given according to the Clubfoot Assessment Protocol-score [24].

### 2.7. Statistical Analyses

The interrater reliability of the radiological measurements was analysed with an interclass correlation coefficient (ICC). An ICC of <0 was classified as poor, 0 < ICC < 0.2 as slight, 0.21 < ICC < 0.4 as fair, 0.41 < ICC < 0.6 as moderate, 0.61 < ICC < 0.8 as substantial, and ICC > 0.81 as almost perfect [25].

To determine the effect of AHDT by means of the 8-plate implantation, the preoperative and postoperative ADTA and dorsiflexion measurements were compared using the paired *t*-test and Wilcoxon Signed-Rank test, respectively. Relations between improvement in ADTA and dorsiflexion measurements were explored by means of Spearman’s rho correlation. In the subgroup of patients with an additional evaluation after at least 1 year after 8-plate removal, differences between the 3 time points were assessed by means of a Friedman test. Wilcoxon Signed-Rank tests with Bonferroni correction were used as post hoc analyses. The effect of timing on changes in ADTA was explored by means of a visual representation.

Statistical analysis was conducted using “IBM SPPS Statistics” software, version 22. A *p*-value < 0.05 was considered statistically significant.

## 3. Results

### 3.1. Patients

Out of 49 patients under 12 years of age who underwent AHDT within our tertiary centre for clubfoot deformities, 32 children (46 relapsed clubfeet) were included in this project, as illustrated in the flowchart in Figure 3. Of these patients, 20 (63%) had unilateral clubfoot, 38% were female and the median age was 7.5 years at the time of 8-plate implantation. For only two children, it was not possible to determine if they underwent Achilles tendon tenotomy as part of the primary Ponseti treatment, as they were initially treated elsewhere. The remaining 30 children (94%) did receive an Achilles tendon tenotomy primarily. The measured alpha angles indicated that all patients had flattening of the talus. Notably, 21 of the 32 patients (66%) received additional treatment before AHDT was indicated (Table 1). Mean treatment duration was 22.1 months (95–CI: 19.3–24.9). In one patient, there were two minor complications, namely superficial wound infection and screw migration. The latter could be accepted without the necessity for treatment adjustments; the infection was successfully treated with oral antibiotics. There were no neurovascular complications.

### 3.2. Additional Interventions

Of the 32 included children, 15 children received additional interventions during 8-plate implantation (Table 2). These interventions included (re-do) Achilles tendon tenotomy, tibialis anterior tendon transfer and derotation osteotomy. During 8-plate removal, an additional 13 patients underwent additional treatment. In the majority of these cases, a TATT was performed (Table 2).

### 3.3. Intrarater Reliability of Radiographic Measurements

The intra-rater reliability for the measurement of the ADTA was almost perfect, with an ICC of 0.930.

### 3.4. Effect of Hemi-Epiphysiodesis Measurements

Dorsiflexion and ADTA for each clubfoot at the different time points are presented in Table 3. For several children, the notated summary of clinical follow-up in the medical records was not detailed enough to reliably extract dorsiflexion or measure ADTA. In all of these children, follow-up was uneventful, and results of AHDT were considered clinically satisfactory. The median ankle dorsiflexion significantly increased during 8-plate treatment (*p* < 0.001), from range −20–5° (median 0°) to range 0–10° (median 0°). Dorsiflexion of 19 patients improved by at least 5 degrees. ADTA significantly improved from 84° (95–CI: 82–85° to 72° (95–CI: 70–74°) during implantation (*p* < 0.001). No significant correlations were found between amount of improvement in ADTA and dorsiflexion.

In the subgroup of patients for whom at least one year after 8-plate removal clinical and radiological assessment was also available, no overall effect of dorsiflexion was found. For example, patient 2 in Table 3 illustrates a significant ADTA loss over time. A clinical decrease in dorsal flexion accompanies an ADTA loss of 9° to 88°. ADTA significantly changed between the three intervals (*p* < 0.001). ADTA significantly decreased during 8-plate treatment (*p* < 0.001), but it significantly increased after at least one year of 8-plate removal compared to the situation at indication for 8-plate removal (*p* < 0.001) (Table 4).

### 3.5. Timing of Hemi-Epiphysiodesis

The age at which the AHDT was performed in this cohort varied between 5 and 12 years. No clear relation between change in ADTA during implantation or amount of rebound after 8-plate removal with age was found (Figure 4A). Changes in ADTA showed a large variation between patients within an age group.

Treatment duration, i.e., number of months the 8-plate was in situ before removal, varied. Again, variation of the accomplished change in ADTA was large, with no clear relation with treatment duration (Figure 4B).

## 4. Discussion

In our patient group, we found a significant decrease in ADTA during temporal AHDT in relapsed, Ponseti-method-treated clubfeet, showing that guided growth is effective in influencing the growth plate and changes the position of the tibial plafond. The disappearance in our patient group of this beneficial change in ADTA over time after removal of the implant is remarkable. After a median follow-up of 20 months, ADTA remodelled to pre-operatively found values. When evaluating the influence of age or duration of the guided growth treatment on change in ADTA, no consistent patterns could be identified. Furthermore, clinical improvement indicated by change in dorsiflexion after guided growth could not unambiguously be quantified in the current cohort.

Radiological improvement quantified with ADTA in relapse clubfoot patients during hemi-epiphysiodesis was previously shown [15,16,17,26]. Although sporadic and minor rebound of ADTA after implant removal was previously reported [15,16,17,26], this is the first study to show severe remodelling at least one year after removal of implants. The distal tibial growth plate closes only in late adolescence [27]. The open growth plate can correct malalignment and is mainly responsible for correcting deformities by asymmetrical growth stimulation. Growth is stimulated on the concave side and inhibited on the convex side [28]. During the hemi-epiphysiodesis, growth is influenced, but after 8-plate removal in the case of open growth plates, one could expect the growth plate to act as before the treatment. This means that remodelling takes place to the old status, where forces in the growth plate are equalised by the above-named mechanism. In our opinion, this phenomenon could be responsible for the rebound effect because during treatment and removal, all growth plates of the patients were still open. This finding could have implications for timing of the procedure. Ideally, AHDT should be performed on older children with approximately two years of growth remaining in the distal tibia. If the growth plate closes after the effect of the hemi-epiphysiodesis, the rebound phenomenon may not be present. If, on the other hand, a hemi-epiphysiodesis cannot be postponed due to patient complaints, the possibility of re-implantation must be considered. In our institution, a clinically significant reduction in dorsal flexion and/or a residual equinus is an indication for considering re-implantation. Of course, there must be growth remaining. Future studies could investigate other factors influencing this rebound phenomenon. If tight structures on the posterior side should play a role, external therapy may be able to neutralise them.

The clinical improvement after guided growth in the distal tibia at longer follow-up in clubfoot children could not unambiguously be quantified in our data. In the complete group, a significant clinical improvement in dorsiflexion was shown till the moment of removal. However, in the smaller subgroup with at least one year of follow-up after implant removal, this improvement in dorsiflexion was not clear. Previous literature showed mostly a positive clinical effect of AHDT on the amount of ankle dorsiflexion [15,16,26], obtaining a plantigrade foot [17] and improved quality of walking according to parents/caregivers [26]. In only one study, a direct relationship is shown between the amount of change in dorsiflexion and a decrease in ADTA [16]. It should be noted that Al-Aubaidi et al. did not find a clinically significant improvement in dorsiflexion in 25 clubfoot patients with progressive equinus relapse previously treated with extensive posteromedial releases [18]. The question remains what the best method is to quantify clinical improvement. A reliable assessment of dorsiflexion is challenging in children with a severe foot deformity [29]. In the current study, all clinical investigations were performed by one senior pediatric orthopaedic surgeon [AB] to minimise variation. Small changes in the position of the foot can, however, have an important impact in clinical function [16]. The difference between a foot in slight equines or slight dorsiflexion is important during gait. It would possibly be more useful to determine if patients achieved a plantigrade foot position [17] or apply extensive gait analyses (e.g., [9,30]) to identify the clinically relevant treatment outcome of AHDT.

It should be noted that the described cohort consists of patients with difficult and heterogeneous clubfoot appearances. Therefore, our conclusions according to functional outcome are mentioned with caution. In line with previous literature, patients often received relapse treatment prior to the hemi-epiphysiodesis and/or the hemi-epiphysiodesis was combined with additional procedures [15,16,17,18]. Our group of patients consisted mostly of referred patients with severe residual clubfoot deformities and flattening of the talus. The aetiology of this talar flattening remains unknown. It may be a pre-existing feature of the clubfoot or related to treatment [31]. Flattening of the talus restricts the movement of the talocrural joint. When non-surgical treatments do not result in improvement, many options involving major surgery are published [32]. In our opinion, ADHT is a reasonable option for these patients.

Before surgical interventions are considered as treatment for relapsed clubfeet, a thorough clinical assessment is necessary [32]. Furthermore, a step-by-step approach, starting with repeated Ponseti casting, should be considered [7,32]. When relapses are again seen in a flexible foot, according to the Ponseti technique itself, a tibialis anterior transfer with or without a re-do Achilles tendon tenotomy should be performed [3]. For older patients with relapsed clubfoot and severe impairment of joint movement, as in flat top talus deformity, less-invasive surgical techniques are preferred to limit joint stiffness in the long term as much as possible [3,33]. Guided growth with its tension band mechanism, introduced with just a small incision, meets these criteria. The post-operative regime after an AHDT includes immediate functional and full weight-bearing instructions, again to prevent any unnecessary joint movement impairment and burden the patient as little as possible. We indicated guided growth when severe functional impairments in combination with complaints in daily life activities existed. It is crucial that these patients are observed for a long time. Patients included in our study will be monitored until the end of growth.

Our study has some limitations. Our patient group is heterogeneous in terms of severity of the deformity and additional treatments besides the hemi-epiphysiodesis, which might influence the clinical outcome. We think, however, that the influence of the deformity severity and additional soft tissue procedures will not influence the growth plate activity that is influenced by AHDT. Also, the technique of minimally invasive surgery to perform AHDT is typically meant for complex cases. Still, these patient factors might complicate the interpretation of the results. Furthermore, although this is the largest cohort described in the literature, the sample size and available follow-up are still limited. Due to the retrospective nature of this study, not all data of all patients was available for analyses. Monitoring of these patients will be continued after the study period. If necessary, additional procedures will be planned. Determining the clinical outcome of hemi-epiphysiodesis purely based on dorsiflexion remains challenging. For future research, we encourage taking the treatment impact on parents and patients into account. Furthermore, more functional and participation measurements could be important to conclude if guided growth is valuable in treating recurrent equinus deformities in clubfeet. If possible, gait analysis could further assist in quantifying the functional status before and after treatment.

In conclusion, AHDT in clubfoot is effective in influencing the growth plate and meets criteria of a less invasive treatment with as little damage as possible. Guided growth by means of hemi-epiphysiodesis should be in the armamentarium in relapse clubfoot with bony impairment after determination of an optimal timing by the treating surgeon. An important finding in this study is the rebound phenomenon. Because rebound of ADTA occurs in children with active growth plates, parents and patients should be informed about this mechanism, which might result in the need for renewed hemi-epiphysiodesis when the procedure had to be used early in childhood. If possible—when indicated—the procedure could be postponed and used in the last phase of growth.

## Figures and Tables

**Figure 1:**
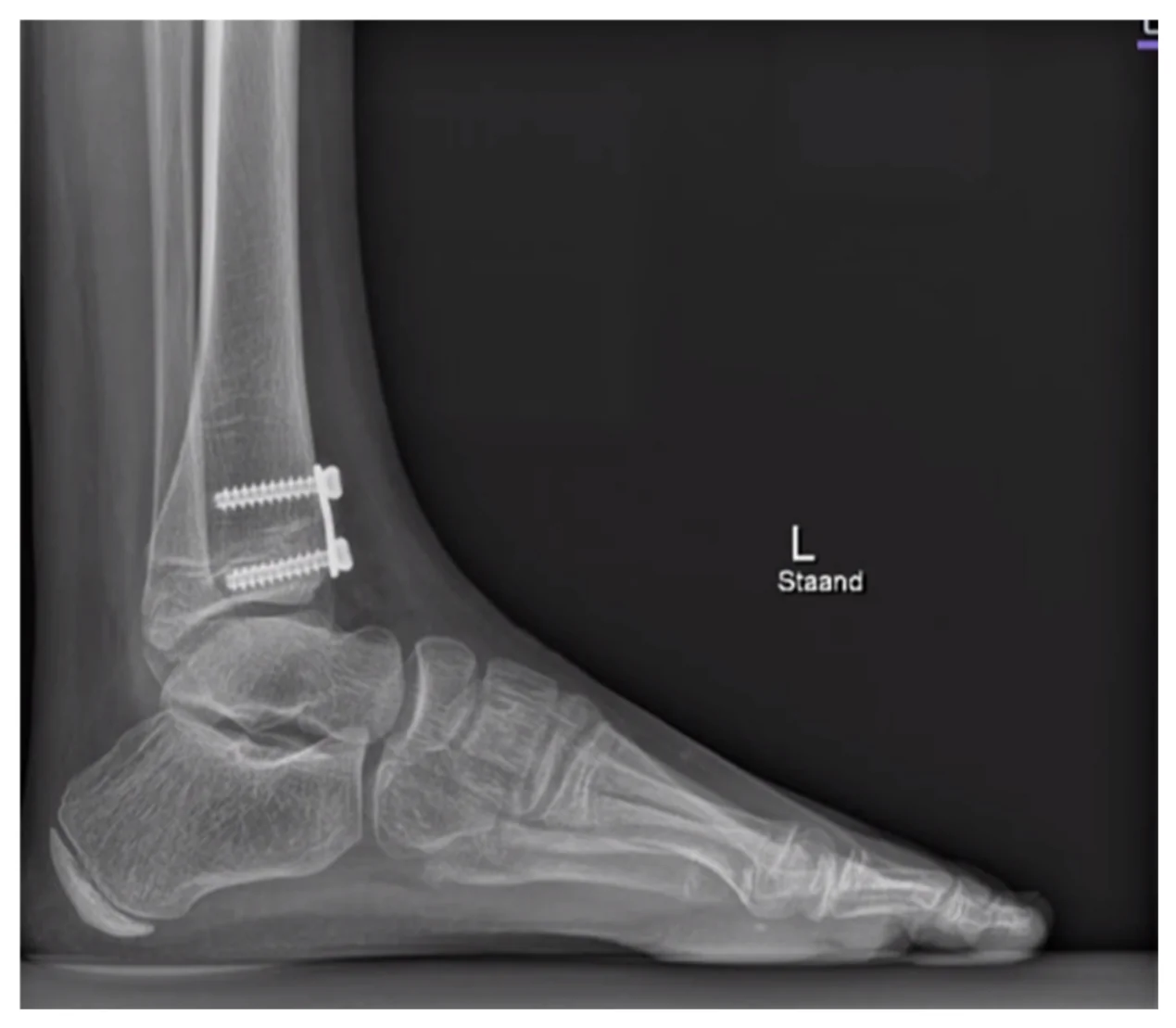
Standard weight-bearing lateral X-rays of the ankle with 8-plate in situ.

**Figure 2:**
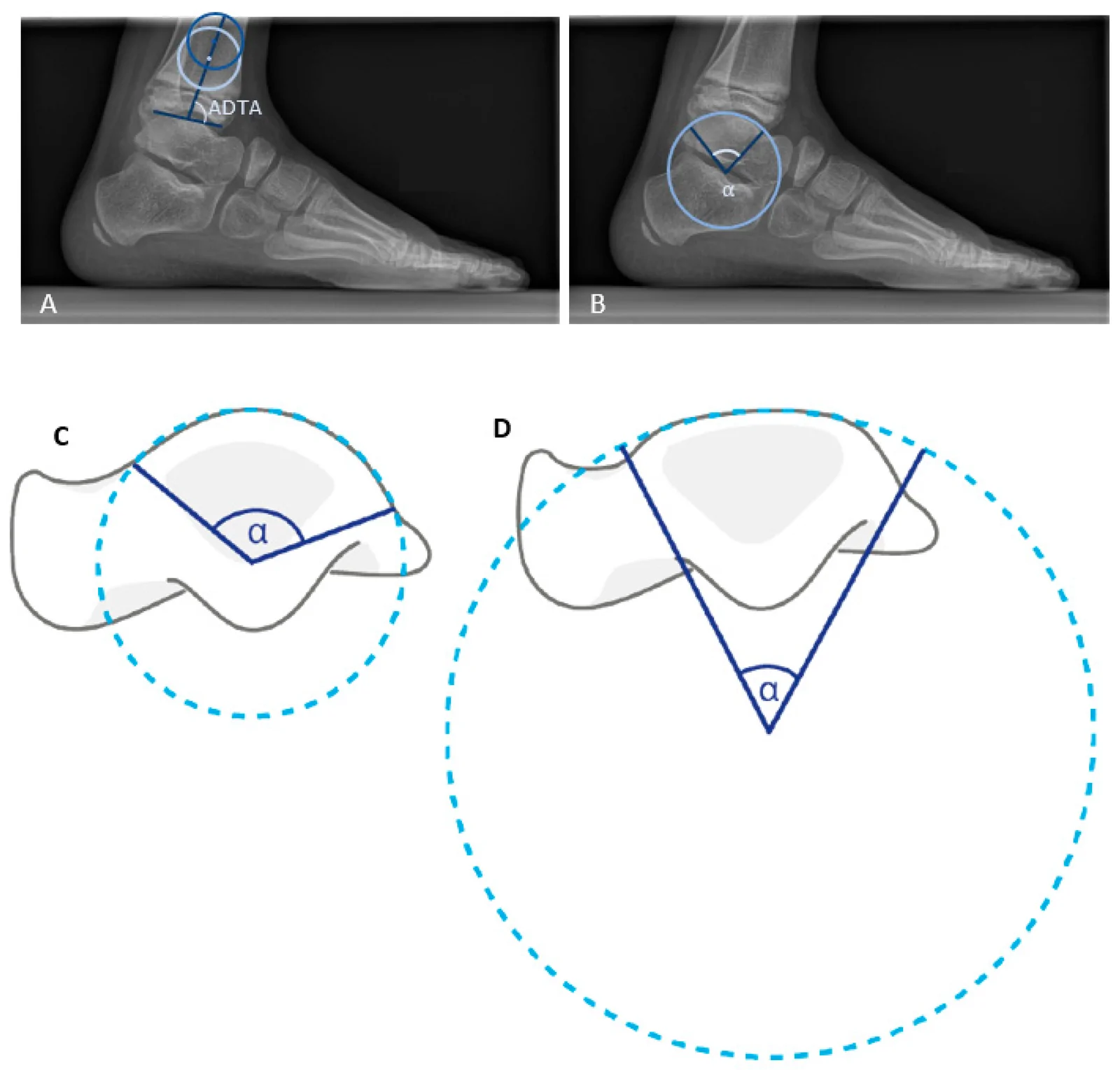
Description of radiographic measurements. (**A**) Lateral weight-bearing radiograph anterior distal tibial angle (ADTA). (**B**) Lateral weight-bearing radiograph of the alpha angle. (**C**,**D**) Illustration of decrease in alpha angle with talar flattening. (**C**) ‘Normal’ shaped talar dome with alpha angle. (**D**) Flattop talar dome with decreased alpha angle.

**Figure 3:**
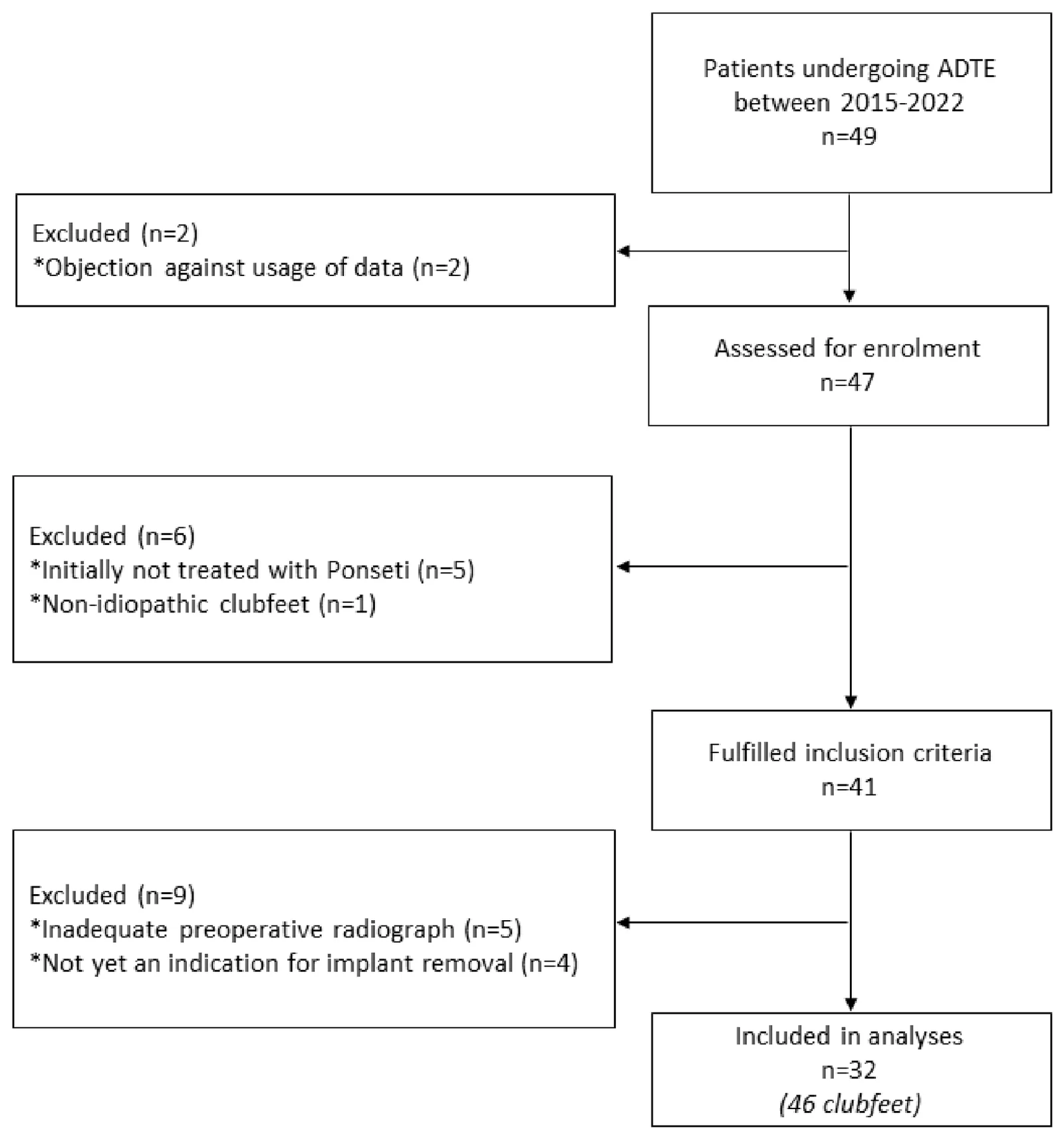
Flowchart of the study population.

**Figure 4:**
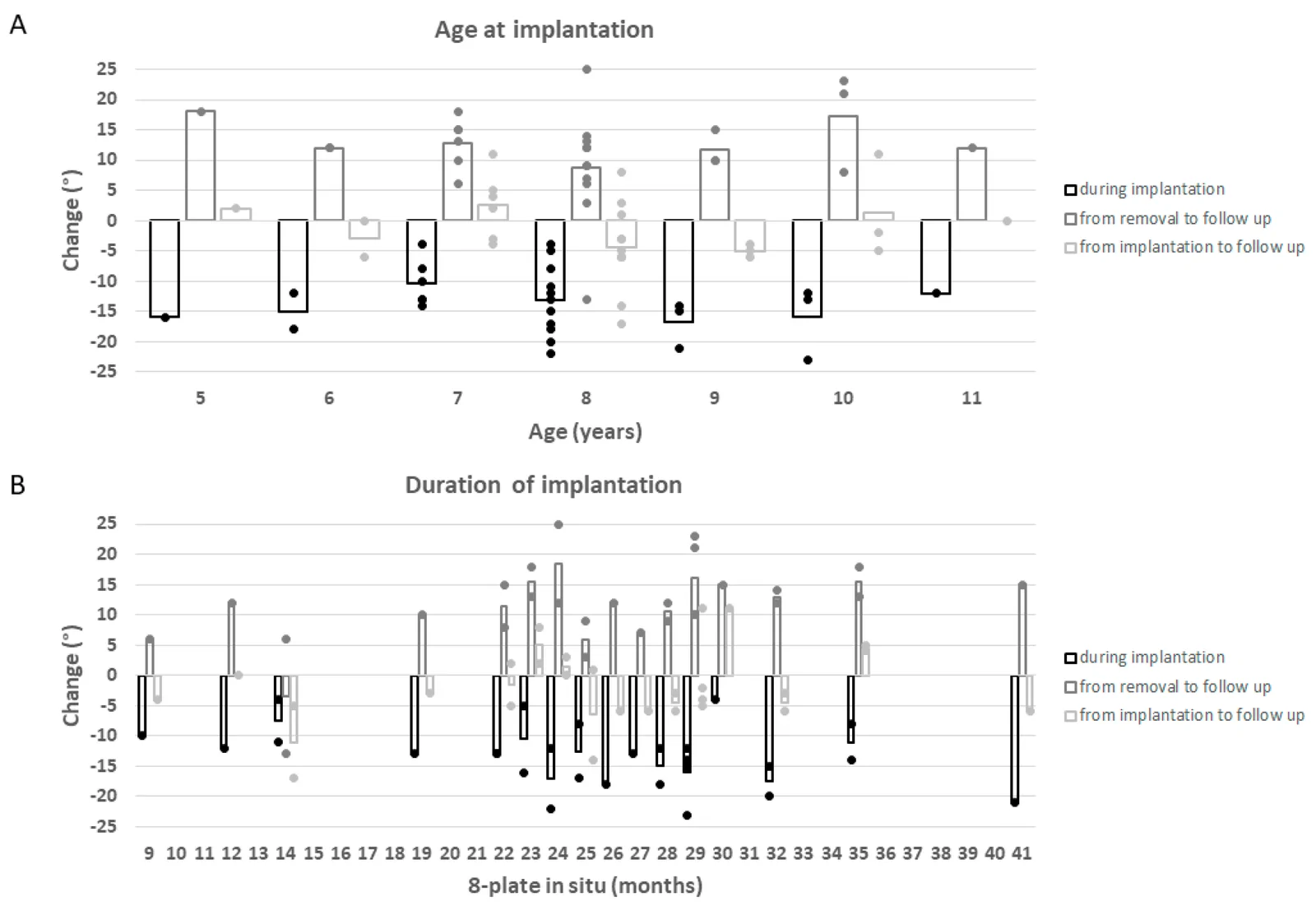
Amount of change accomplished during implantation, after implant removal and between initial implantation of 8-plate and final follow-up. Bars show mean change in ADTA for each category, and dots represent results for a single patient. (**A**) Change in ADTA versus age at implantation. (**B**) Change in ADTA versus duration of 8-plate in situ.

**Table 1:** Patient characteristics.

	Sex	Side	Age at Time of Implantation (Years Old)	Alpha Angle (°) on Pre-Operative Radiograph	Additional Treatment Before 8-Plate Implantation
1	Male	Right	7	58.4	TATT
2	Female	Bilateral	8	42.4/32.0	Bilateral TATT
3	Female	Right	7	49.3	None
4	Male	Left	10	60.2	None
5	Male	Bilateral	6	42.8/56.1	None
6	Female	Left	9	28.0	Additional bracing
7	Male	Right	8	59.5	TATT
8	Male	Bilateral	7	82.6/73.8	None
9	Male	Bilateral	9	84.5/79.4	None
10	Male	Left	8	25.6	TATT
11	Female	Bilateral	8	109.3/91.1	Bilateral TATT + PMR
12	Male	Right	11	45.3	None
13a *	Female	Left	5	33.3	PMR
13b *	Female	Left	10	66.6	8-plate (see case 13a)
14	Female	Left	5	15.0	Re-do AT
15	Male	Right	6	16.0	Additional casting
16	Female	Bilateral	7	36.3/41.9	Additional casting + bracing
17	Male	Bilateral	7	27.1/33.6	Bilateral TATT + re-do AT
18	Male	Bilateral	8	49.7/30.0	Bilateral additional casting + TATT
19	Female	Bilateral	8	95.0/81.4	None
20	Female	Left	10	43.9	TATT
21	Male	Right	7	53.6	Additional bracing + re-do AT
22	Male	Bilateral	6	37.5/55.0	Bilateral TATT
23	Female	Right	7	88.0	Additional bracing
24	Female	Left	11	*-*	None
25	Male	Right	11	49.1	TATT + twice re-do AT
26	Female	Right	8	80.8	Additional casting + additional bracing
27	Male	Left	6	83.2	None
28	Male	Bilateral	8	75.9/70.2	None
29	Male	Right	6	60.1	Additional bracing
30	Male	Bilateral	7	82.8/72.0	None
31a °	Female	Left	7	72.9	None
31b °	Female	Right	7	53.0	None
32	Male	Right	9	*-*	TATT

* Represents 1 patient who had a re-do 8-plate implantation in the same foot. ° Represents 1 patient who has bilateral clubfoot with same 8-plate implantation date; however, with a different date of implant removal. Abbreviations: PMR = Posteromedial Release; AT = Achilles tenotomy; TATT = tibialis anterior tendon transfer; - = Missing.

**Table 2:** Additional interventions during follow-up period.

			Additional Interventions During 8-Plate Implantation		Additional Interventions During 8-Plate Removal	
Study No.	Clubfoot	Time (Months) 8-Plate In Situ	Side	Additional Intervention	Side	Additional Intervention
1	Right	29.6	None		Right	ATT
2	Bilateral	27.7	None		None	
3	Right	15.8	None		Right	TATT
4	Left	22.2	None		Left	TATT
5	Bilateral	20.1	None		Bilateral	TATT
6	Left	12.8	Left	Derotation osteotomy	Right	Epiphysiodesis foot
7	Right	23.6	None		Right	TATT
8	Bilateral	23.6	Bilateral	TATT	None	
9	Bilateral	29.1	Bilateral	TATT	None	
10	Left	26.6	None		None	
11	Bilateral	24.9	Bilateral	TATT	Bilateral	Epiphysiodesis distal fibula
12	Right	23.6	None		None	
13a *	Left	23.0	Left	TATT	None	
13b *	Left	24.0	Left	AT	None	
14	Left	17.2	None		Left	TATT
15	Right	18.2	Right	TATT + AT	None	
16	Bilateral	6.8	Bilateral	TATT	None	
17	Bilateral	21.7	None		None	
18	Bilateral	32.4	None		None	
19	Bilateral	14.0	Left Right	ATT + TAT ATT	Right	Osteotomy Mt1-5
20	Left	29.4	None		None	
21	Right	34.7	Right	ATT	None	
22	Bilateral	26.0	Bilateral	Derotation osteotomy distal tibia	Bilateral	Removal derotation osteotomy plate
23	Right	34.0	Right	TATT	None	
24	Left	11.4	None		None	
25	Right	32.3	None		None	
26	Right	13.1	None		None	
27	Left	13.8	None		None	
28	Bilateral	22.5	Bilateral	ATT	Right	TATT
29	Right	11.7	None		Right	TATT
30	Bilateral	18.7	None		None	
31a °	Left	9.4	None			TATT
31b °	Right	18.3	None		None	
32	Right	40.5	Right	TATT	None	

* Represents 1 patient who had a re-do 8-plate implantation in the same foot. ° Represents 1 patient who has bilateral clubfoot with same 8-plate implantation date; however, with a different date of removal. Abbreviations: ATT = Achilles tendon tenotomy; TATT = tibialis anterior tendon transfer.

**Table 3:** Results per clubfoot.

		Dorsiflexion			ADTA		
Study No.	Side	Preoperative Measurement	Indication for 8-Plate Removal	At Least One Year Follow-Up	Preoperative Measurement	Indication for 8-Plate Removal	At Least One Year Follow-Up
1	R	0	0	*-*	76	72	87
2	L	0	10	−10	91	79	88
2	R	5	0	0	94	76	88
3	R	−5	0	0	81	73	*-*
4	L	0	5	15	83	70	78
5	L	0	0	0	84	76	*-*
5	R	0	0	10	83	72	*-*
6	L	0	0	*-*	86	63	*-*
7	R	5	0	−5	89	67	92
8	L	0	0	*-*	86	63	*-*
8	R	0	5	*-*	86	71	*-*
9	L	*-*	*-*	10	84	69	79
9	R	*-*	*-*	10	86	72	82
10	L	−20	5	0	84	71	78
11	L	0	*-*	10	80	72	81
11	R	0	*-*	0	81	64	67
12	R	−10	5	0	83	71	83
13a *	L	−5	0	*-*	79	63	81
13b *	L	−20	−5	*-*	71	67	*-*
14	L	0	0	*-*	87	73	*-*
15	R	0	0	*-*	88	72	*-*
16	L	0	10	*-*	85	81	*-*
16	R	0	10	*-*	86	86	*-*
17	L	0	0	−10	80	67	82
17	R	0	0	−10	77	*-*	87
18	L	0	0	0	85	70	82
18	R	0	0	−10	88	68	82
19	L	0	10	0	84	73	79
19	R	0	*-*	10	84	80	67
20	L	*-*	5	10	72	60	83
20	R	−10	5	0	85	62	83
21	L	5	0	0	77	69	82
21	R	5	0	0	81	67	85
22	R	−10	5	0	82	64	76
23	L	5	*-*	*-*	77	75	*-*
24	R	*-*	*-*	*-*	88	69	*-*
25	R	0	10	*-*	80	73	*-*
26	L	0	0	10	80	*-*	97
27	L	0	10	10	89	71	*-*
27	R	−10	10	0	91	97	*-*
28	R	0	-	0	75	70	83
29	L	0	10	*-*	84	72	84
30	R	0	10	*-*	86	73	83
31a °	L	−5	0	0	84	74	80
31b °	R	0	*-*	10	84	83	*-*
32	R	*-*	0	*-*	94	73	88

* Represents 1 patient who had a re-do 8-plate implantation in the same foot. ° Represents 1 patient who has bilateral clubfoot with same 8-plate implantation date; however, with a different date of removal. Abbreviations: R = Right; L = Left; - = Missing.

**Table 4:** Functional and radiographic outcomes in 3 measurement intervals, presented as median [range] or mean [95–Confidence Interval].

	Preoperative Measurement	Indication for 8-Plate Removal	At Least One Year Follow-Up	Significance
Dorsiflexion n = 22 feet	0° [range: −20–5°]	0° [range: 0–10°]	0° [range: −10–15°]	*p* = 0.076
ADTA n = 27 feet	83° [95–CI: 81–85°]	70° [95–CI: 68–72°]	82° [95–CI: 79–84°]	*p* < 0.001 *

* Post hoc analyses indicated a significant decrease in the ADTA from preoperative measurement to the indication for 8-plate removal and a significant increase in ADTA from indication for 8-plate removal and follow-up.

## Data Availability

The data presented in this study are available on request from the corresponding author. The data are not publicly available due to privacy and ethical restrictions.
